# Smartphone Use and Social Media Involvement in Young Adults: Association with Nomophobia, Depression Anxiety Stress Scales (DASS) and Self-Esteem

**DOI:** 10.3390/ijerph21070920

**Published:** 2024-07-15

**Authors:** Elissavet Vagka, Charalambos Gnardellis, Areti Lagiou, Venetia Notara

**Affiliations:** 1 Department of Public and Community Health, School of Public Health, University of West Attica, 12243 Athens, Greece; elvagka@uniwa.gr (E.V.); alagiou@uniwa.gr (A.L.); vnotara@uniwa.gr (V.N.); 2 Department of Fisheries and Aquaculture, School of Agricultural Sciences, University of Patras, 30200 Messolonghi, Greece

**Keywords:** smartphone use, social media, young adults, nomophobia, depression, anxiety, stress, self-esteem

## Abstract

Excessive smartphone use and dependence on social media give rise to multiple issues that negatively affect the overall well-being of individuals. Nomophobia is characterized as a “digital disease” due to the unlimited use of smartphone devices. The aim of this study is to examine smartphone use and social media involvement in association with nomophobia and psychological traits (i.e., depression, anxiety, stress, and self-esteem) of young adults. A cross-sectional investigation was conducted on 1408 Greek undergraduate students aged 18 to 25 years, using an anonymous self-reported questionnaire. Study data were collected through the Nomophobia Questionnaire (NMP-Q), Depression Anxiety Stress Scale (DASS-21), and Rosenberg Self-Esteem Scale (RSES). Participants’ social media engagement was summarized through four variables: use of social media applications, number of friends, number of followers, and number of messages exchanged daily on social media. High levels of nomophobia were associated with high engagement in social media and, thereby, with a high number of friends. The same applies to participants with high/normal self-esteem compared to those with low. Regarding stress, anxiety and depression were not associated with digital network use, while elevated stress and anxiety seemed to play a negative role in the number of online followers. In addition, high levels of anxiety were correlated to an increased number of messages exchanged through social media applications.

## 1. Introduction

Human nature has consistently exhibited the tendency to implement methods to facilitate daily life [1]. As language evolved, one pivotal advancement in this concern was the development of communication techniques, with phone devices providing the main means of contact. Over time, the evolution of communication technology transitioned from wired phones to the contemporary smartphone, which is one of its notable achievements.

No recent innovation has had such a catalytic effect on consumers’ lives as smartphones. According to the Pew Research Center (2021) [2], 96% of young adults in the United States were in possession of a smartphone, while a substantial one-third of total consumer expenditures, over USD 1 trillion, were conducted through mobile platforms [3]. The number of users worldwide has increased from 3.678 billion in the year 2016 to 6.928 billion in 2023 [4], while an increase to 7.7 billion users is estimated for the year 2027 [5]. In Greece, according to the most recent findings, the vast majority of the population (97%) use internet applications, with smartphones ranking first among consumer preferences (97%), followed by laptops (51%). Furthermore, 88% appear to use social media, while they report spending an average of more than 1.5 h daily engagement with these applications/platforms [6].

Contemporary societies demonstrate a strong tendency to use technological and digital advancements [7]. Nowadays, smartphones with more advanced features (cameras, gaming, data storage, document processing) play a pivotal role in daily living. Findings from the EU Kids Online 2020 reported that the majority of children in Europe 9–16 years old opted for smartphones as their preferred means of accessing the internet, given the assurance of continual connectivity and accessibility [8]. However, this convenience is not offered without consequences; unlimited and unregulated or obsessive use of smartphones has been associated with the development of behavioral issues and mental health disorders [9].

Recent research has highlighted that excessive smartphone use gives rise to a multitude of issues, such as dependence on social media and problematic internet use. These issues negatively affect an individual’s psychological, social, academic, and professional well-being [10,11]. Frequent engagement with smartphones, including activities such as checking text messages or emails, accessing social networks, and internet browsing, has been associated with adverse effects on various aspects of well-being, which encompass disruptions in sleep patterns, high levels of stress and anxiety, decreased levels of physical activity and diminished academic performance [12].

Almost from the beginning of the current century, there has been a significant increase in internet use, with websites serving as invaluable sources of information worldwide. Certain websites and applications continue to streamline daily tasks, facilitating uninterrupted communication with family and friends regardless of time or location. Social media includes a range of internet-based applications enabling the creation and interchange of user-generated content [13]. Social media has evolved into a crucial component for communicating with others, sharing personal content, and accessing news and entertainment. A considerable percentage of the younger population is deeply engaged with social media platforms. Approximately 90% of young adults in developed countries use online networking sites, with the majority visiting them at least once a day [14].

Nomophobia is characterized as a “digital disease” due to the excessive use of smartphone devices [15]. The term is derived from the abbreviation NO-MObile-PHOne- phoBIA and manifests as fear and anxiety induced by the absence of a mobile phone [16]. Nomophobia reflects an addictive pattern affecting young adults worldwide [17,18,19]. It seems to be a phobia of the modern age as a result of an individual’s interaction with ever-evolving technology [16]. The inability to use smartphone functions can negatively affect psychosocial health and lead to long-term negative feelings [20]. Moreover, recent research has identified a correlation between nomophobia and the existence of depression, anxiety, and stress [21,22]. Demographic characteristics, such as gender, seem to be associated with an increased risk of developing nomophobia, with women being more prone [23,24,25].

In addition, depression and eating concerns emerge as significant negative consequences of excessive social media usage [14]. Regular engagement with social media may foster a perception that others lead happier, more socially connected lives, thereby heightening feelings of social isolation and ultimately contributing to depression [26]. Particularly, Facebook, Instagram, and Twitter stand out as the most extensively used social media platforms among young adults [27]. The substantial time spent on these platforms not only reduces their physical activity but also establishes specific cognitive patterns, potentially leading to psychological challenges and, eventually, depression [14].

Various studies have demonstrated an association between social media and anxiety. It has been estimated that 45% of British adults become anxious when they feel unable to access social networks and their emails [28]. Furthermore, higher levels of anxiety, stress, and depression among students classified as internet addicts compared to normal users were observed [29]. Moreover, a high frequency of daily social media use was associated with increased levels of dispositional anxiety and potential anxiety disorder [30].

The use of social media can act as a stressor or perpetuate negative self-perception, particularly when individuals encounter unfavorable feedback from peers or are engaged with adverse social comparisons [31]. The results of an Iranian study indicated a connection between the use of online social media and stress among male users, while a notable association was observed in the presence of depression, anxiety, and stress among female users [32]. Additionally, the American Psychiatric Association (2017) stated that 43% of Americans habitually check their email and virtual networks, resulting in behaviors associated with stress [33].

A recent study conducted in Greece estimated that 73.1% of the participants used more than 5 different social media platforms daily. The most frequent platforms were Facebook/Messenger (98.0%) and Instagram (88.2%), followed by Viber (78.0%), YouTube (67.5%), and Pinterest (36.9%). Furthermore, the results suggested a link between psychological distress and social media addiction, indicating that individuals with increased distress levels were more likely to report increased levels of addiction to social media applications. This observation was notably significant across all three negative psychological states, i.e., depression, anxiety, and stress [34].

Moreover, social media use is associated with low self-esteem in young adults. Self-esteem is defined as “a subjective evaluation of one’s own value or worth” [35]. Several studies with a wide age range of participants, from adolescents to older adults, demonstrated a negative correlation between addictive social media use and self-esteem [36,37,38,39]. Furthermore, social media use was related to increased life satisfaction and self-esteem levels [40], while frequent and positive feedback on social media profiles also showed higher users’ self-esteem levels [41]. It seems that the association between social media and individuals’ self-esteem depends on (a) the way in which the user interacts with social media content [36,37,38,42], (b) the content’s nature [43], and (c) individuals’ unique characteristics and sensitivities [44,45].

In a study at the University of Pennsylvania, students were asked to reduce the use of Facebook, Instagram, and Snapchat to 10 min per platform/day. The results indicated that constraining social media use to approximately 30 min daily could result in substantial enhancements in overall well-being [46].

There is a lack of evidence in the literature regarding the association of smartphone use and social media with nomophobia and negative emotional states such as depression, anxiety, and stress among young adults. The manifestation of these emotional states (i.e., mood disorder, worry or fear, and response to stressful conditions) have adverse impacts on daily living. Therefore, regardless of the literature gap, the current study tried to investigate this interesting and potentially emerging issue among young adults. It is an attempt to outline, from an exploratory perspective and without the assumption of specific causal models to be confirmed, the association among DASS, nomophobia, self-esteem, and social media in Greece. In this context, the research hypothesis of the study concerns the potential relationship between participants’ social media engagement as an outcome and nomophobia, anxiety, depression, stress, and self-esteem levels as predictors. Under this spectrum, the research questions are formulated as follows:

Q1: How do young adults’ levels of nomophobia differ in terms of sociodemographic variables?
Q2: Is there a relationship between participants’ social media checking, the number of friends, followers, and texting/day to nomophobia, anxiety, depression, stress, and self-esteem levels?
Q3: Is there an association between participants’ social media checking, the number of friends, followers, and texting/day with nomophobia levels, DASS components, and self-esteem due to sociodemographic variables?

As indicators of the participants’ involvement with social media, four measures were used as dependent variables: checking social media from a smartphone, number of friends, followers, and texting/day. The nomophobia, anxiety, depression, stress, and self-esteem levels were used as independent variables. It was considered that the four variables defining social media engagement include the more specific measures mentioned in the literature (mainly that of marketing), such as the number of likes and reactions, comments, shares and reposts, time spent on content, etc. [47]. The number of friends and followers or the messages exchanges/day are considered to be directly related to the psychological traits of the participants and are not mediators like the online functions such as comments, shares, likes, etc.

## 2. Materials and Methods

### 2.1. Subjects

A cross-sectional study with a sample of 1408 young adults was conducted in Athens, Greece, including 1099 (71.7%) females and 399 (28.3%) males. The participants were recruited from six different faculties of the University of Western Attica (School of Public Health, School of Health and Care Sciences, School of Engineering, School of Administrative, Economics and Social Sciences, School of Food Sciences, and School of Applied Arts and Culture) and Public Vocational Training Institutes, which are located in the greater area of Athens. It is necessary to mention that the University of West Attica is the third largest in Greece in terms of the number of students. The mean age of the sample was 20.7 years; 31.5% were employees, and 25.8% were living alone. Inclusion criteria were defined as follows: (a) belonging to the age group of 18–25 years, (b) owning a smartphone, and (c) completing the informed consent form.

### 2.2. Study Procedure

Data collection involved the dissemination of a self-administered online questionnaire. Prior to completing the study questionnaire, participants were required to provide informed consent. The questionnaire was designed using the Google Forms application, and the corresponding web link was made available through the e-class platform of the courses during the 2020–2021 academic year. Participation was entirely voluntary, devoid of any associated incentives or penalties. The study’s researcher conveyed the study’s purpose to participants through the Microsoft Teams platform. Furthermore, efforts were made to ensure that all essential information was adequately communicated and accessible during the questionnaire completion process. The study was authorized by the University of West Attica’s Research Committee (14/21-09-2020) and was conducted in compliance with the Declaration of Helsinki (1989).

### 2.3. Data Collection Tools

The data collection tool included five components: (a) Nomophobia Questionnaire (NMP-Q), (b) the Depression Anxiety Stress Scale (DASS-21), (c) the Rosenberg Self-Esteem Scale (RSES), (d) sociodemographic variables, such as age, gender, participants’ educational level, year of study and parents’ educational background, and (e) questions regarding social media, such as ‘’What applications do you have on your smartphone?’’, “How many friends do you have on Facebook, Messenger or Games?”, and “How many followers do you have on Facebook, Instagram and Twitter?”.

#### 2.3.1. Nomophobia Questionnaire (NMP-Q)

The NMP-Q, developed and validated by Yildirim and Correia (2015), serves as a tool for assessing nomophobia. It consists of 20 items and is divided into four subscales: (a) not being able to communicate (i.e., “I would feel nervous because I would not be able to receive text messages and calls”), (b) losing connectedness (i.e., “I would be uncomfortable because I could not stay up-to-date with social media and online networks”), (c) not being able to access information (i.e., “I would feel uncomfortable without constant access to information through my smartphone”), and (d) giving up convenience (i.e., “Running out of battery in my smartphone would scare me”). Participants rate each item on a 7-point Likert scale ranging from 1 (totally disagree) to 7 (totally agree). The total score ranges from 20 to 140, with higher scores indicating a more pronounced experience of nomophobia [16]. The NMP-Q underwent adaptation and validation for use in the Greek language. Both exploratory and confirmatory factor analyses were conducted, indicating a four-factor structure that aligns with the original instrument (Cronbach-a values for each factor were: (a) 0.936, (b) 0.895, (c) 0.867, and (d) 0.854, similar to the original NMP-Q of (a) 0.939, (b) 0.827, (c) 0.819, and (d) 0.874, respectively). The total scale revealed a high internal consistency, like the original NMP-Q (Cronbach-a 0.945 for both questionnaire versions) [48].

#### 2.3.2. Depression Anxiety Stress Scale (DASS-21)

The DASS-21 is a condensed version of Lovibond and Lovibond’s (1995) original DASS-42 questionnaire [49]. The tool is structured into three discrete subscales, each comprising seven items, designed to assess depression (depression scale), anxiety (anxiety scale), and stress (stress scale). The depression scale evaluates symptoms such as distress, hopelessness, diminished interest/engagement, and anhedonia (questions 3, 5, 10, 13, 16, 17, and 21, i.e., “I couldn’t seem to experience any positive feeling at all”). The anxiety scale targets aspects including autonomic nervous system arousal, musculoskeletal system effects, state anxiety, and subjective experiences related to anxiety (questions 2, 4, 7, 9, 15, 19, and 20, i.e., “I felt I was close to panic”). Moreover, the stress scale aims to assess a state of chronic arousal and tension characterized by difficulties in relaxation, overstimulation, heightened aggression, irritability, and impatience (questions 1, 6, 8, 11, 12, 14, and 18, i.e., “I tended to over-react to situations”).

Participants were required to indicate the severity of each symptom experienced during the preceding week using a 4-point Likert scale (0 = not applicable at all, 3 = very applicable or most of the time), assigning scores ranging from 0 to 3 points for each statement. To compute the total negative affective state score for each participant, the scores for all items are summed. The final score on the DASS-21 questionnaire is then obtained by multiplying this total score by 2. Depression scores are categorized as follows: normal (0–9), mild (10–13), moderate (14–20), severe (21–27), and extremely severe (28 and above). Anxiety scores are classified as normal (0–7), mild (8–9), moderate (10–14), severe (15–19), and extremely severe (20 and above). Stress scores fall into the following categories: normal (0–14), mild (15–18), moderate (19–25), severe (26–33), and extremely severe (34 and above).

The Cronbach’s alphas of the study DASS subscales were 0.90, 0.88, and 0.88 for depression, anxiety, and stress, respectively, suggesting a high internal consistency of the scales.

#### 2.3.3. Rosenberg Self-Esteem Scale (RSES)

The RSES comprises ten items aimed at evaluating an individual’s overall self-esteem by encompassing both positive and negative self-perceptions. Renowned for its reliability and precision in quantitatively assessing self-worth, this scale employs a Likert scale for responses, ranging from 0 to 3, where 0 corresponds to “strongly disagree” and 3 to “strongly agree”. Examples of scale sentences are: “I feel that I have a number of good qualities” and “I generally tend to feel like I’m a failure”. Noteworthy is the reverse scoring of items 3, 5, 8, 9, and 10. The total score is derived by summing the Likert values, with a score below 15 indicating low self-esteem, a score between 15 and 25 indicating a normal level of self-esteem, and a score surpassing 25 indicating high self-esteem. While originally devised to gauge self-esteem among high school students, this scale has been applied across diverse age categories, including adults [50]. The study’s Cronbach’s alpha coefficient for the RSES was 0.81, indicating a high degree of internal consistency similar to previous studies [51].

### 2.4. Data Analysis

Data analysis was conducted using descriptive statistics, univariate techniques, and multivariate regression analysis. Absolute and relative frequencies (%) were given for nominal and ordinal variables, while continuous variables were presented by their mean values and quartiles. Comparisons between nominal variables were undertaken through the χ^2^ test and for continuous variables by means of Kruskal–Wallis analysis of variance and *t*-test. Internal consistency of nomophobia and DASS components was evaluated through Cronbach’s alpha. To avoid the problem of small frequencies that appear in certain DASS categories, the latter were consolidated with others. This contributes to homogenizing the score distributions and makes the results more robust and coherent. In addition, it increases the power of statistical analyses. The new emerging categories, along with their bounds for each component of DASS, are Anxiety, normal (0–7), moderate = mild/moderate (8–14), severe = severe/extremely severe (15 and above); Depression, normal (0–9), moderate = mild/moderate (10–20), severe = severe/extremely severe (21 and above); Stress, normal (0–14), moderate = mild/moderate (15–25), severe = severe/extremely severe (≥26).

In the regression models, total nomophobia was transformed to a 100-point scale so that relative changes in the variable values were interpreted as percentages. Where deemed necessary for the purposes of the analysis, the nomophobia score was classified into three escalating categories (mild nomophobia = 21–59 of total NMP-Q score, moderate nomophobia = 60–99 of NMP-Q, and severe nomophobia ≥ 100 of NMP-Q). Rosenberg’s scale was used to determine low (RSES < 16) and normal values (RSES = 16–25) of self-esteem.

Participants’ social media involvement was assessed using four variables: whether they use their mobile phones to check social networks, the number of friends and followers they have on social networks, and the number of messages they receive or send on a daily basis. The four variables were used as response variables in a set of regression models with predictors the components of DASS (anxiety, stress, and depression), nomophobia, and self-esteem. Due to high existing correlations between the components of DASS, models were run separately for each component. In total, a bunch of 12 regression models were assessed: three logistic regression models for checking social networks through smartphones (one for each DASS component) and corresponding linear regression models (separately for depression, anxiety, and stress) for the number of friends, followers, and messages received/sent per day.

All previous models were adjusted for gender, age, working status, residency, nationality, and parents’ education of participants. Statistical calculations were performed using SPSS version 28 statistical software (SPSS Inc., IBM Corp, Armong, NY, USA).

At this point, it should be emphasized that the approach to data analysis is exploratory and not confirmatory (e.g., through structural equation models). The lack of literature regarding the formulation of a model that defines the relationship between social media and negative emotional states or nomophobia made it necessary to first describe this relationship (if indeed it exists). The use of structural equation models could be a confirmatory procedure if the relationship is first defined descriptively. The regression models used in the analysis were simple in their linear expression and easy to interpret. Furthermore, they were assessed separately for each parameter of the DASS to avoid collinearity problems that exist between depression, anxiety, and stress.

## 3. Results

Of the participants, 71.7% were women and 28.3% were men. Nomophobia ranged from mild in 24.1% of them, moderate in 57%, and severe in 18.9%. Levels of nomophobia appeared higher in women compared to men, i.e., 21.2% of women showed severe nomophobia compared to 13.1% of men, while in the mild level, men showed higher percentages (30.8% versus 21.4% of women). Regarding the other characteristics, milder levels of nomophobia appeared in those who were employed versus non-employed, i.e., 28.8% vs. 21.9% in mild nomophobia and 14.6% vs. 20.9% in severe, and slightly milder in those over 21 years compared to 18–21 yrs: 25.8% vs. 22.4% in mild nomophobia and 17.1% versus 20.6% in severe (Table 1).

A high percentage of participants (61%) were identified with severe anxiety, while the percentage of those who showed severe depression and stress was much lower (28.8% and 16.9%). However, moderate depression and stress were observed in 31.5% and 18.4% of them, respectively. In addition, most of the study subjects, 81.5%, had normal or high self-esteem, with only 18.5% of them being identified as low (Table 2).

Significant differences in checking social media were found in terms of nomophobia levels and self-esteem. The higher the nomophobia, the higher the percentages reported regarding checking social networks via smartphone. Those with severe nomophobia reported high rates of using their phone to check social media (91.7%) compared to those with moderate and mild levels (81.1% and 71.1%, respectively) (*p* < 0.001). Furthermore, people with normal to high self-esteem seem to make more use of social networks via smartphone, 82.7% vs. 74.7% of low self-esteem (*p* = 0.003). Differences were also found in the number of friends and followers, primarily in relation to participants’ self-esteem and secondarily to nomophobia. Participants with normal/high self-esteem tend to have more online friends and followers than others. Their average number of friends and followers was 1057 and 650 compared to 835 and 548, respectively, for participants with low self-esteem (both *p* values < 0.015). Differences were also found between those with severe nomophobia and those identified with high stress in terms of the number of followers, i.e., 737 followers for those with severe nomophobia versus 628 and 554 for those with moderate and mild, respectively (*p* = 0.042). Regarding stress, participants with severe stress levels also had a greater number of followers compared to the rest, i.e., 746 vs. 526 and 631 of those with moderate and low stress (*p* = 0.026) (Table 3).

All previous relationships became clearer when controlling for gender, age, working status, residency, nationality, and education of students’ parents. Testing was performed using a number of logistic and linear regression models with dependent variables checking social networks through mobile phones, number of friends, followers, and messages received/sent per day.

From the logistic models (one for each component of DASS), significant differences in checking social media through smartphones were found in relation to participants’ nomophobia and self-esteem scaling. For every percentage point increase in nomophobia score, the odds ratio of using a smartphone to check social networks increases by about 3% (95% CI: 1.02, 1.4), while people with low self-esteem lagged behind others in checking social media by about 50% (apparently due to their reduced number of friends and followers on social platforms). Differences in the same direction appeared regarding the number of the participants’ online friends. Participants with elevated nomophobia tended to have a greater number of friends, approximately 3.5 friends for each percentile increase in the nomophobia scale (3.6, 95% CI: 0.3, 5.8 in the anxiety scale model and 3.6, 95% CI: 0.4, 7.0 in the depression model). Similarly, regarding the self-esteem scale, participants with low self-esteem had fewer online friends by about 200 (in the anxiety scale model by 216.3, (95% CI: −386.2–46.3), in the stress model by 235.1, (95% CI: −407.8,−62.3) and in the depression model by 194.4, (95% CI: −379.7, −9.2).

Regarding the number of followers, differences were found between participants with increased nomophobia (the number of followers increased by about 4 followers for every percentage point increase in nomophobia), among participants with low self-esteem (people with low self-esteem have about 100 followers less than others) but also between men and women (women have about 135 more followers than men). In addition, in the topic of online followers, an important role seems to be played by the anxiety and stress variables. Participants with moderate and severe anxiety appeared to have a lower number of followers than normal ones, 147.5 and 106.6 fewer followers, respectively. Similarly, participants with moderate stress appeared to have a lower number of online followers (112.8 fewer followers).

Finally, an increased number of messages per day was observed among participants with higher anxiety levels (moderate and severe). They exchange about 3 more messages per day than the rest. Nomophobia and self-esteem did not seem to play a significant role in the number of messages exchanged by the participants (Table 4).

## 4. Discussion

In the contemporary digital age, the prevalence of social media platforms has witnessed a remarkable upsurge, and in many cases, they have substituted traditional face-to-face interactions. Social media, in digital form, has emerged as a dynamic and interactive tool facilitating multifaceted communication and dissemination of diverse information, ideas, and expressions. The evolving landscape of social media use highlights its transformative influence on communication patterns and the broader societal structure [52]. The findings of the study indicated that social media involvement was closely connected with and affected by self-esteem and nomophobia. Much weaker is the relationship between social media and DASS parameters. Although these relationships did not lead to causal interpretations, the identified associations were robust and controlled for several potential confounding parameters.

The results of this study indicated that nearly all individuals exhibited some degree of nomophobia, with the highest proportion exhibiting a moderate level. Several studies also reported moderate nomophobia levels, while recent systematic reviews demonstrated the prevalence of nomophobia among young adults, particularly among university students [53,54,55].

Regarding sociodemographic characteristics, lower levels of nomophobia appeared in the participants aged 21 years or older. Similarly, a study conducted on Peruvian medical students observed lower nomophobia levels in participants aged ≥21 years of age compared to those under 21 [17], while another one stated that for people older than 20 years, nomophobia tends to decrease with age [18]. However, other studies found no differences in the prevalence of nomophobia according to age, at least among young adults [56,57]. In terms of gender differences, women were at greater risk of being identified with severe nomophobia symptoms compared to men. The scientific literature regarding gender disparities in nomophobia levels remains controversial, with some studies aligning with the findings of the present study [23,24], while others failed to identify significant differences [58,59]. These gender variations may be attributed to distinct patterns of smartphone use, with men primarily using smartphones for professional purposes and women for interpersonal communication [60]. Non-working young adults were more likely to show higher levels of nomophobia than working ones.

Moreover, the findings identified a high prevalence of severe anxiety among participants, with a notably lower proportion experiencing severe depression and stress. Furthermore, most of the study subjects exhibited normal to high levels of self-esteem, as similarly observed in university students [61].

Significant differences in checking social media through smartphones were found in terms of nomophobia levels and self-esteem. Participants with severe nomophobia reported using their phones to check social media. Gezgin et al. (2018) [62] mentioned that social media users checked their smartphones more often than others, while the increased use of social media platforms was consistent with the increased levels of nomophobia. A recent study investigating the relationship between nomophobia and the extensive use of social networking applications, feelings of loneliness, and loss of control, demonstrated a significant association between social media addiction and nomophobic manifestations [63]. Within the context of relevant studies, individuals with higher levels of nomophobia demonstrated increased use of social media applications [64,65]. In the same direction, another study showed that social media addiction was linked to students’ nomophobia levels [66].

The thorough investigation of the relationship between self-esteem and social media use, after controlling for several sociodemographic parameters, showed that participants with normal to high self-esteem were more likely to use social networks via smartphone. A systematic review stated that social networking application use was linked to higher levels of life satisfaction and self-esteem [40]. Nevertheless, recent findings demonstrated a negative correlation between social media and individuals’ self-esteem [35,38,39]. In contrast, Marengo et al. (2021) indicated that individuals’ self-esteem increased when they had frequent and intensive positive feedback on their social media profiles [41].

Discrepancies were also observed in the number of friends and followers, mainly in relation to self-esteem and, subsequently, to nomophobia. Participants with normal to high self-esteem had more friends and followers on social media platforms compared to others. A related study disclosed that the number of friends exhibited a positive correlation with self-esteem in the long term, and it was regarded as a positive component of one’s constructive use of Facebook [67]. Nevertheless, although social media, on certain occasions, has the potential to foster the development of friendships, research indicates that excessive use has a detrimental effect on self-esteem and overall life satisfaction [68].

Regarding nomophobia, participants with increased nomophobia levels tended to have a greater number of social media friends and followers. Recent studies observed that individuals with a higher number of digital friends and followers, compared to face-to-face friendships, exhibited increased levels of nomophobic manifestations [69]. Despite the scarcity of literature evidence, the prevailing picture is that social media overuse is consistent with high nomophobia levels [63,64,70].

Exploring the number of online followers, anxiety and stress levels seemed to play an important role. Participants identified with moderate to severe anxiety or moderate stress appeared to have a lower number of online followers than others. In the same line, Sharma et al. (2022) revealed that in the case where an individual had a reduced number of followers, likes, and comments, there was a higher probability that this individual would perceive it as a lack of social acceptance from their peers. Seeking approval through social comparison might eventually lead to the emergence of mental health issues such as anxiety [71].

The findings of the study indicated that social media involvement was closely connected with and affected by self-esteem and nomophobia. Much weaker is the relationship between social media and DASS parameters. Although these relationships did not lead to causal interpretations, the identified associations were robust and controlled for several potential confounding parameters. The regression models used, by their linear expression, defined the dependent and explanatory variables of the analysis and, therefore, predetermined the direction of the possible causal relationships. Checking social media by smartphone, number of digital friends and followers, and number of messages exchanged via social networks were defined as outcome variables for social media engagement. The explanatory variables, depression, anxiety, stress, self-esteem, and nomophobia, were modeled as potentially causally related to the involvement with digital networks. Obviously, the inverse relationships could also be tested, e.g., the number of digital friends or followers as a cause of depressive or anxiety disorder (rather exaggerated as a single unidirectional hypothesis). It is reasonable to assume that depression, stress, anxiety, or self-esteem predate digital media use. However, they may be degenerated by the increased use of digital media. This would define a bidirectional relationship, which, however, would be the subject of a specific analysis, e.g., using structural equation models. However, in this case, we would have to use exogenous variables (not presented in our data) and make specific assumptions regarding the relationships of the studied variables, which do not seem to exist in the literature so far. Moreover, it must be pointed out that the number of digital friends or followers as a cause for nomophobia is rather meaningless for obvious reasons. In any case, the cross-sectional nature of the study makes it very difficult to investigate bidirectional causal relationships between the DASS parameters, self-esteem, nomophobia, and social media involvement. A longitudinally designed epidemiological study with these as key variables could illuminate the problem in more detail.

Smartphones constitute a prominent tool utilized by the younger generation. Hence, those devices play a crucial role in shaping the communication patterns of young individuals, mainly on social media platforms. Moreover, a large percentage of young people, exceeding 80%, reported feeling deeply connected to their social friends through online platforms [72]. This status has notably affected the mental health of numerous young adults, leading to adverse psychological consequences. With the escalating ubiquity of information and communication technologies, additional research is required, such as qualitative and observational studies, to delve deeper into the real-world impact of social media on user behavior. These diverse research methods can provide a comprehensive understanding of how social media shapes the daily lives of its users.

In addition, the results indicate a need for awareness and prevention programs from young ages that will contribute to the promotion of controlled and responsible social media use. Likewise, it is necessary to provide counseling and behavior modification therapy among problematic social media users. By implementing multifaceted interventions, social media’s adverse effects can be mitigated.

Nevertheless, the benefits of online platforms, such as media literacy, creativity, self-expression, sense of belonging, and civic participation, should not be underestimated [73].

### Limitations

Understanding the limitations of the current study is vital during the analysis of the findings. Due to the cross-sectional nature of the investigation, it is rather difficult to generalize the results. Additionally, social media utilization is not equal around the world and can be affected by factors such as geographic location, education, and economic status. Individuals’ social media use, the content they share, and how they interact with others varies cross-culturally. An important limitation is also that the study was conducted among students from one University and Post-Secondary Vocational Training schools from Attica prefecture, despite the fact that the University of West Attica is the third largest in Greece in terms of students’ number and faculties. Moreover, while the use of self-reported questionnaires is a methodological restriction (however, they remain the most widely used assessment tools to date), the significance of such data remains important. In any case, the cross-sectional nature of the study makes it very difficult to investigate bidirectional causal relationships between the DASS parameters, self-esteem, nomophobia, and social media involvement. A longitudinally designed epidemiological study with these as key variables could illuminate the problem in more detail.

## 5. Conclusions

The relationships identified in the present study can be summarized as follows. High levels of nomophobia were associated with high engagement with social media and, consequently, with a high number of friends. The same applies to participants with high/normal self-esteem versus those with low. Stress, anxiety, and depression were not associated with digital network use, while only high stress and anxiety seemed to play a negative role in the number of online followers. In addition, moderate and severe anxiety was correlated to an increased number of digital messages.

Scientific research demonstrates that daily social interactions among young people mainly occur in virtual environments rather than in person. For the new generation of young adults, online social media has been a constant presence, shaping their digital interactions as the standard mode of communication. It is important that health education and health promotion programs should focus on individuals’ overall well-being, starting at a young age, with a specific emphasis on promoting safe usage of social media platforms.

## Figures and Tables

**Table 1:** Distribution of study subjects according to sociodemographic characteristics and nomophobia levels.

			Nomophobia					
	Total		Mild		Moderate		Severe	
	N	%	N_1_ 339	% 24.1	N_2_ 803	% 57.0	N_3_ 266	% 18.9
Gender								
Men	399	28.3	123	30.8	224	56.1	52	13.1
Women	1009	71.7	216	21.4	579	57.4	214	21.2
Age								
18–20	711	50.5	159	22.4	405	57.0	147	20.6
21+	697	49.5	180	25.8	398	57.1	119	17.1
Work								
No	964	68.5	211	21.9	552	57.3	201	20.9
Yes	444	31.5	128	28.8	251	56.5	65	14.6
Residency								
Alone	363	25.8	107	29.5	180	49.6	76	20.9
With parents	1045	74.2	232	22.2	623	59.6	190	18.2
Nationality								
Greek	1319	93.9	314	23.8	757	57.4	248	18.8
Others	85	6.1	24	28.2	43	50.6	18	21.2
Father education								
University	474	33.7	130	27.4	266	56.1	78	16.5
Other	934	66.3	209	22.4	537	57.5	188	20.1
Mother education								
University	604	42.9	142	23.5	356	58.9	106	17.5
Other	804	57.1	197	24.5	447	55.6	160	19.9

**Table 2:** Distribution and descriptive measures of study subjects according to DASS components and Rosenberg Self-Esteem Scale.

	Total		Men		Women	
	N	%	N	%	N	%
Anxiety						
Normal	254	18.0	80	20.1	174	17.2
Moderate	295	21.0	99	24.8	196	19.4
Severe	859	61.0	220	55.1	639	63.3
Stress						
Normal	911	64.7	283	70.9	628	62.2
Moderate	259	18.4	61	15.3	198	19.6
Severe	238	16.9	55	13.8	183	18.1
Depression						
Normal	559	39.7	157	39.3	402	39.9
Moderate	443	31.5	136	34.1	307	30.4
Severe	406	28.8	106	26.6	300	29.7
Self-esteem						
Low	261	18.5	74	18.5	187	18.5
Normal/High	1147	81.5	325	81.5	822	81.5
	Mean	SD	Mean	SD	Mean	SD
Anxiety	9.4	5.6	8.6	5.4	9.7	5.6
Stress	6.1	5.6	5.2	5.2	6.5	5.7
Depression	7.3	5.7	6.9	5.5	7.4	5.8
Self-esteem	20.6	6.4	20.8	6.6	20.5	6.4

Anxiety: normal (0–7), moderate = mild/moderate (8–14), severe = severe/extremely severe (15 and above). Depression: normal (0–9), moderate = mild/moderate (10–20), severe = severe/extremely severe (21 and above). Stress: normal (0–14), moderate = mild/moderate (15–25), severe = severe/extremely severe (26 and above). Self-esteem: Low (<15), Normal/High (15 and above).

**Table 3:** Checking social media through smartphone (yes/no) and mean number of friends, followers, and messages/day according to DASS, nomophobia, self-esteem, and gender *.

	Checking Social Media			Friends					Followers					Messages/Day				
			*p*		Percentiles			*p*		Percentiles			*p*		Percentiles			*p*
	N	% **	Value	Mean	25	50	75	Value	Mean	25	50	75	Value	Mean	25	50	75	Value
Anxiety			0.412					0.857					0.603					0.121
Normal	199	78.3		1060	216	787	1436		707	200	455	857		22	6	20	40	
Moderate	243	82.4		998	203	704	1420		576	182	450	750		25	8	25	40	
Severe	702	81.7		1009	200	692	1424		627	180	450	800		25	6	22	40	
Stress			0.698					0.997					0.026					0.230
Normal	742	81.4		1017	203	700	1432		631	177	450	800		25	7	22	40	
Moderate	213	82.2		913	200	720	1350		526	150	435	714		23	4	20	40	
Severe	189	79.4		1123	200	660	1442		746	238	499	922		26	7	21	40	
Depression			0.390					0.430					0.663					0.427
Normal	461	82.5		1077	206	730	1462		665	182	460	844		24	6	20	40	
Moderate	362	81.7		1001	220	700	1407		621	200	450	750		25	7	25	40	
Severe	321	79.1		948	197	655	1400		596	177	440	800		25	4	23	40	
Nomophobia			<0.001					0.474					0.042					0.354
Mild	241	71.1		1007	158	600	1412		554	120	400	760		25	6	25	40	
Moderate	659	82.1		983	200	660	1420		628	190	450	780		24	6	20	40	
Severe	244	91.7		1125	380	856	1444		737	250	500	915		25	6	23	40	
Self-esteem			0.003					0.001					0.015					0.340
Low	195	74.7		835	114	600	1204		548	139	417	728		24	4	20	40	
Normal/High	949	82.7		1057	235	714	1440		650	200	460	810		25	7	20	40	
Gender			0.046					0.972					<0.001					0.766
Men	311	77.9		1014	200	696	1400		521	130	358	663		25	6	20	40	
Women	833	82.6		1016	209	700	1439		674	226	484	835		25	6	20	40	

* Tested by means of *χ*^2^ for checking social media, Kruskall–Wallis analysis of variance for DASS and nomophobia, and *t*-test for self-esteem and gender. ** Percentage of participants who check social media through smartphones for each category of DASS, self-esteem, and gender variables.

**Table 4:** Logistic and linear regression derived coefficients * for models with response variables: Checking social media via smartphone (yes/no), number of friends, followers, and messages/day. Explanatory variables in the models are anxiety, stress, depression (DASS), nomophobia, and self-esteem. Due to high existing correlations between the components of DASS, models were run separately for each component.

	Checking Social Media ^1^			Friends ^2^			Followers ^2^			Messages/Day ^2^		
	*OR*	95% CI *OR*	*p*	*B*	95% CI *B*	*p* Value	*B*	95% CI *B*	*p* Value	*B*	95% CI *B*	*p* Value
			Value									
Anxiety												
Anxiety—Moderate ^a^	1.16	(0.75, 1.79)	0.513	−71.3	(−275.1, 132.6)	0.493	−147.5	(−271.2, −23.7)	0.020	3.1	(−0.2, 6.3)	0.063
Anxiety—Severe ^a^	1.08	(0.74, 1.58)	0.685	−28.8	(−204.0, 147.5)	0.749	−106.6	(−213.6, 0.4)	0.051	3.2	(0.3, 6.0)	0.028
Nomophobia	1.03	(1.02, 1.04)	<0.001	3.6	(0.3, 5.8)	0.033	4.2	(2.2, 6.2)	<0.001	−0.1	(−0.1, 0.1)	0.687
Self-esteem—Low ^b^	0.49	(0.34, 0.69)	<0.001	−216.3	(−386.2, −46.3)	0.013	−109.8	(−213.1, −6.6)	0.037	−1.8	(−4.5, 0.9)	0.197
Gender—Women	1.13	(0.83, 1.15)	0.440	−10.2	(−152.6, 132.2)	0.889	135.1	(48.7, 221.6)	0.002	−0.2	(−2.4, 2.1)	0.880
Stress												
Stress—Moderate ^a^	1.04	(0.71, 1.53)	0.849	−73.2	(−244.6, 98.2)	0.403	−112.8	(−216.9, −8.8)	0.034	−1.8	(−4.5, 1.0)	0.208
Stress—Severe ^a^	0.73	(0.49, 1.10)	0.131	138.3	(−46.7, 323.3)	0.143	91.8	(−20.5, 204.1)	0.109	1.2	(−1.8, 4.1)	0.431
Nomophobia	1.03	(1.02, 1.04)	<0.001	3.1	(−0.3, 6.4)	0.072	3.6	(1.6, 5.7)	<0.001	−0.1	(−0.2, 0.5)	0.851
Self-esteem—Low ^b^	0.52	(0.36, 0.74)	<0.001	−235.1	(−407.8, −62.3)	0.008	−125.0	(−229.8, −20.1)	0.020	−1.3	(−4.1, 1.4)	0.347
Gender—Women	1.13	(0.84, 1.53)	0.421	−8.4	(−150.7, 133.8)	0.908	138.5	(52.1, 224.9)	0.002	−0.1	(−2.4, 2.2)	0.937
Depression												
Depression—Moderate ^a^	0.88	(0.62, 1.24)	0.454	−46.4	(−199.4, 106.6)	0.552	−42.5	(−135.6, 50.6)	0.371	1.6	(−0.8, 4.0)	0.199
Depression—Severe ^a^	0.82	(0.55, 1.20)	0.304	−47.0	(−223.1, 129.1)	0.601	−61.9	(−168.9, 45.2)	0.257	2.3	(−0.6, 5.1)	0.116
Nomophobia	1.03	(1.02, 1.04)	<0.001	3.6	(0.4, 7.0)	0.028	4.1	(2.1, 6.1)	<0.001	−0.1	(−0.2, 0.1)	0.793
Self-esteem—Low ^b^	0.54	(0.37, 0.79)	0.002	−194.4	(−379.7, −9.2)	0.040	−92.1	(−204.7, 20.6)	0.109	−2.3	(−5.3, 0.7)	0.128
Gender—Women	1.12	(0.83, 1.51)	0.453	−9.5	(−151.8, 132.7)	0.896	135.3	(48.8, 221.8)	0.002	−0.1	(−2.4, 2.2)	0.921

* Adjusted for age, working status, residency, nationality, and parents’ education. ^1^ Logistic regression models with response variable checking social media via smartphone (yes/no). ^2^ Linear regression models with response variables number of friends, followers, and messages/day. ^a^ Reference category is Normal. ^b^ Reference category is Normal/High.

## Data Availability

Data are available upon request.
